# Cacao Polyphenol-Rich Dark Chocolate Intake Contributes to Efficient Brain Activity during Cognitive Tasks: A Randomized, Single-Blinded, Crossover, and Dose-Comparison fMRI Study

**DOI:** 10.3390/nu16010041

**Published:** 2023-12-21

**Authors:** Akihiro Sasaki, Eriko Kawai, Kyosuke Watanabe, Emi Yamano, Chisato Oba, Kentaro Nakamura, Midori Natsume, Kei Mizuno, Yasuyoshi Watanabe

**Affiliations:** 1 Laboratory for Pathophysiological and Health Science, RIKEN Center for Biosystems Dynamics Research, 6-7-3 Minatojima-Minamimachi, Chuo-ku, Kobe 650-0047, Hyogo, Japan; kawai@respiratorycontrol.com (E.K.); kyosuke.watanabe@dragon.kobe-u.ac.jp (K.W.); emi.yamano@riken.jp (E.Y.); keimizuno@riken.jp (K.M.); yywata@riken.jp (Y.W.); 2 RIKEN Compass to Healthy Life Research Complex Program, 6-7-3 Minatojima-Minamimachi, Chuo-ku, Kobe 650-0047, Hyogo, Japan; 3 Center for Health Science Innovation, Osaka Metropolitan University, 3-1 Ofukacho, Kita-ku, Osaka 530-0011, Osaka, Japan; 4 Food Microbiology Research Laboratories, R&D Division, Meiji Co., Ltd., 1-29-1 Nanakuni, Hachioji 192-0919, Tokyo, Japan; kentarou.nakamura@meiji.com (K.N.); midori.natsume@meiji.com (M.N.)

**Keywords:** dark chocolate, cacao polyphenols, cognitive function, functional magnetic resonance imaging

## Abstract

Cacao polyphenol-enriched dark chocolate may have beneficial effects on human health, such as facilitating maintaining good performance in long-lasting cognitive tasks. This study examined the effects of dark chocolate intake on improving brain function during cognitive tasks using functional magnetic resonance imaging (fMRI). In this randomized, single-blinded, crossover, and dose-comparison study, 26 healthy middle-aged participants ingested dark chocolate (25 g) either with a low concentration (LC) (211.7 mg) or a high concentration (HC) (635 mg) of cacao polyphenols. Thereafter, their brain activities were analyzed during continuous and effortful cognitive tasks relevant to executive functioning using fMRI in two consecutive 15 min sessions (25 and 50 min after ingestion). We observed significant interaction effects between chocolate consumption and brain activity measurement sessions in the left dorsolateral prefrontal cortex and left inferior parietal lobule. After HC chocolate ingestion, these areas showed lower brain activity in the second session than in the first session; however, these areas showed higher activity in the second session after LC chocolate ingestion. These results suggest that cacao polyphenol-enriched dark chocolate enhances the efficient use of cognitive resources by reducing the effort of brain activity.

## 1. Introduction

Polyphenols are found in foods and beverages such as coffee, red wine, tea, and certain fruits and vegetables [1]. They have antioxidant properties [1,2,3] and may have beneficial effects on human health. Dark chocolate is rich in cacao polyphenols, which contain abundant epicatechins, catechins, and their oligomeric procyanidins, and are associated with the improvement in endothelial vascular function [4,5,6]. The association between cacao polyphenols and cognitive function has also been investigated. Some behavioral studies have shown that a single ingestion of cacao polyphenols improves cognitive performance [7,8], inhibits increased subjective fatigue [8], and helps to maintain concentration and mental stability during long-lasting cognitive tasks [9]. Chronic intake of cacao polyphenols has also been reported to improve cognitive performance in terms of verbal learning, memory, and attention in young adults [10].

Cacao polyphenols have also been reported to improve blood flow in both the peripheral and central nervous systems and to enhance brain activity related to cognitive performance. A previous study reported increased cerebral blood volume in the hippocampal dentate gyrus after 3 months of continuous high-cacao polyphenol food consumption in older adults [11]. The study also showed an improvement in memory performance compared with that when consuming a low-cacao polyphenol food [11]. In another study, a single dose of a cacao polyphenol-enriched cocoa drink resulted in increased central nervous system blood flow, with a peak increase in blood flow at 2 h after ingestion and a return to baseline at 6 h [12]. Some neuroimaging studies have shown that brain activity relevant to memory and/or attention tasks, particularly in the prefrontal and parietal regions, is improved by consuming high cacao flavanols sub-chronically (for approximately 1 week) [12,13]. A more recent study reported that acute cacao polyphenol consumption improved reaction time in the Flanker test and increased brain activity in the supramarginal and inferior frontal gyri in individuals with type 1 diabetes and matched controls [14]. Another study has reported that dark chocolate intake decreased the delta and theta absolute power and increased the alpha and beta absolute power of electrical activity in most brain regions [15]. These findings suggest that even a single ingestion of cacao polyphenol contributes to brain functions associated with cognitive tasks.

We have also previously investigated how an acute intake of chocolate with high cacao polyphenol content affects cognitive function during tasks that require high cognitive effort in healthy individuals. In that study, we used a “traffic light test” as a continuous and effortful task that demands high cognitive efforts with inhibition to a Stroop stimulus and selective attention to a disturbing stimulus, and we showed that a single intake of chocolate with a high concentration of cacao polyphenols (HC chocolate) helped maintain cognitive performance and concentration during continuous and effortful tasks compared with chocolate with a low concentrations of cacao polyphenols (LC chocolate) [16]. However, it was unclear whether the acute intake of HC chocolate increases brain activity efficiency during continuous and effortful tasks. In this study, we measured brain activity during a similar traffic light test and examined whether the difference in brain activity between early and late time points after ingestion depended on the cacao polyphenol concentration.

## 2. Materials and Methods

### 2.1. Participants

After eligibility screening, 55 adult volunteers aged 30–49 years participated in this study between August 2020 and April 2021. The study was conducted over seven study periods, with screening tests performed at the beginning of each study period to select participants for the following 2-day functional magnetic resonance imaging (fMRI) study. Participants were selected in order of earliest application from those who satisfied the inclusion criteria and did not violate the exclusion criteria (listed in Table S1). Finally, 33 participants (18 males and 15 females) participated in the fMRI study. The study was conducted in compliance with national legislation and the Code of Ethical Principles for Medical Research Involving Human Subjects of the World Medical Association (Declaration of Helsinki) and registered in the University Hospital Medical Information Network (UMIN) Clinical Trials Registry (No. UMIN000041636). The study protocol was approved by the Ethics Committee of the RIKEN Center for Biosystems Dynamics Research (RIKEN BDR), Kobe, Japan (approval no. RIKEN-K2-2020-01) and Meiji Co. Ltd., Hachioji, Japan (approval no. 187). All participants provided written informed consent before enrollment in this study.

### 2.2. Study Design

This was a single-blinded (investigators and assessors were blinded), randomized, and dose-comparison study with a two-phase crossover design conducted at RIKEN BDR between September 2020 and June 2021. The target number of cases (N = 30) was determined by a power analysis using the results of cognitive function tests from a previous study [16] to determine the number of participants for which the significance level alpha and power of the test would be 0.05 and 0.8, respectively. Participants were assigned by a researcher who was not involved in data collection and analysis according to the dynamic assignment procedure. Dynamic allocation is a method of sequentially determining groups to which new participants are assigned, reflecting the balance of background factors among previously assigned groups. Participants were then assigned to either group A or B based on age, sex, and body mass index (BMI); participants in group A consumed HC chocolate on day 1 and LC chocolate on day 2, whereas participants in group B consumed LC chocolate on day 1 and HC chocolate on day 2. This study followed the CONSORT guidelines for reporting randomized crossover trials [17].

**Figure 1:**
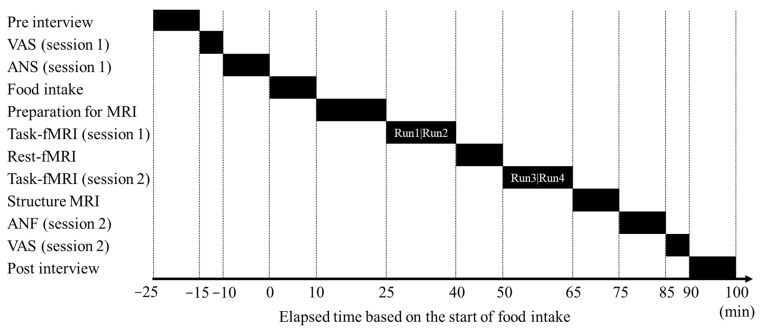
Gantt timeline depicting the daily schedule followed during the study. Each task-fMRI session (1 and 2) comprised two MRI scans: Run 1 and Run 2 constitute session 1, and Run 3 and Run 4 constitute session 2. ANF, autonomic nervous system function assessment; fMRI, functional magnetic resonance imaging; MRI, magnetic resonance imaging; VAS, visual analog scale.

The study schedule is presented in Figure 1. First, the participants were briefed about the study; we then confirmed that they were in good physical condition and had not exercised excessively or consumed chocolate, cocoa, or alcohol on the day before the study or any meal, snack, or beverage other than water for 6 h before the test began.

Next, the participants completed a subjective feeling questionnaire and autonomic nervous system function assessment. They then ingested the test food during the 10 min test food intake period. Subsequently, the participants were transferred to the MRI room and prepared for scanning; 25 min after the start of the test food ingestion, the first-task fMRI (session 1) scan was conducted for 15 min. Following a 10 min resting-state fMRI scan, a second task fMRI (session 2) scan was conducted for 15 min. A structural MRI (sMRI) scan was then conducted, after which the participants completed another subjective feeling questionnaire and autonomic nervous system function assessment. Finally, we assessed the participants’ physical condition.

### 2.3. Test Food

We used two types of chocolates containing different concentrations of cacao polyphenols (Table 1), which had the same compositions as the chocolates used in a previous study [16]. One serving of HC and LC chocolate contained 635.0 mg and 211.7 mg of cacao polyphenol, respectively. The LC chocolate was used for the control condition, and it was equivalent to commercially available, ordinary chocolate with a minimum amount of cacao polyphenols that was lower than the effective cocoa polyphenol amount reported in a previous systematic review of cognitive function [18]. Both test foods were manufactured by the R&D Division of Meiji Co., Ltd. (Hachioji, Japan) for this study. The contents of each chocolate were known only to the manufacturers, and the allocation was concealed from the investigators. To ensure blinding, white chocolate was not used as a control because of its appearance and the possible effects of milk protein.

### 2.4. Analysis Groups

Of the 55 participants, 33 were randomly divided into two groups (Figure S1). As5 participants discontinued or dropped out, 28 of the 33 participants completed the study. The per-protocol analysis set was defined as participants who completed the full study schedule and did not meet any of the following analytic exclusion criteria: (i) test food intake rate < 90%, (ii) behavior that undermined the reliability of test results (such as dozing off during the test). Consequently, 26 participants were included in the per-protocol analysis. In total, 14 (93.3%) of the 15 participants in group A that consumed HC chocolate first and 12 (92.3%) of the 13 in group B that consumed LC chocolate first completed the study (Figure S1).

### 2.5. Outcome Measures

The primary outcome measures were brain activity parameters during the cognitive tasks from sessions 1 and 2. The secondary outcome measures included cognitive task performance from sessions 1 and 2 and subjective feelings and autonomic nervous system function before and after intake.

### 2.6. MRI

#### 2.6.1. Data Acquisition

All images were obtained using a 3T MR scanner (Siemens Prisma, Erlangen, Germany) with a 32-channel RF-receiver head coil and the harmonization protocol developed by the Brain/MINDS-Beyond Human Brain MRI Project (https://hbm.brainminds-beyond.jp/documents/protocol.html, accessed on 1 August 2020). sMRI images were obtained using 3D T1-weighted (T1w) MPRAGE and 3D T2-weighted (T2w) SPACE with 0.8 mm isotropic resolution and pre-scan normalization. Two runs of resting-state fMRI (rest-fMRI) and four runs of task fMRI (task-fMRI) were performed using gradient-echo echo-planar imaging (EPI) with the following parameters: number of volumes = 320 for rest-fMRI and 528 for task-fMRI, voxel size = 2.0 mm isotropic resolution, 72 slices without gap, TR = 750 ms, TE = 36.2 ms, FA = 55°, and multi-band factor = 8, phase encoding direction AP and PA for each run. A single-band reference image was also obtained at the beginning of each run, and two runs of spin-echo EPI scans were used for distortion correction.

#### 2.6.2. Data Processing

All imaging data in the Digital Imaging and Communications in Medicine standard format were converted to the Neuroimaging Informatics Technology Initiative (NIFTI) format and organized into participants-based folder structures using BCILDCMCONVERT (https://github.com/RIKEN-BCIL/BCILDCMCONVERT, accessed on 1 August 2020). The NIFTI data were preprocessed according to the Human Connectome Project (HCP) minimal preprocessing pipeline (HCP-MPP) [21]. Imaging data were analyzed using HCP pipelines (https://github.com/Washington-University/Pipelines, accessed on 1 May 2019), Connectome Workbench (https://github.com/Washington-University/workbench, accessed on 1 May 2019), FreeSurfer 5.3 (https://surfer.nmr.mgh.harvard.edu/), Functional Magnetic Resonance Imaging of the Brain Software Library (FSL) 6.0.1 (www.fmrib.ox.ac.uk/fsl, accessed on 1 August 2020), and MATLAB (http://www.mathworks.com, accessed on 1 August 2020). The sMRI images were corrected for gradient nonlinearity, readout distortion, and bias field; aligned to AC-PC native space; and registered to MNI152 space using the FSL’s FNIRT. Native space images were used to generate individual white and pial surfaces [21] using the FreeSurfer pipeline and HCP-MPP. In the PostFreeSurfer pipeline, the native-mesh surfaces of individuals were registered using a multi-modal surface matching (MSM) algorithm [22] with MSMSulc to the Conte69 folding-based template [23] and resampled to a 32k mesh in each hemisphere. Cortical myelin content was estimated by dividing the T1w image by the T2w image and mapping the Gaussian mid-thickness weighted values of voxels between the outer and inner cortical surfaces onto the 32k cortical mid-thickness surface [21,24,25]. The cortical thickness generated by FreeSurfer was also mapped onto the 32k cortical surfaces. The cleaned time-series fMRI data were used to apply weighted regression of spatial independent component-analysis templates and align onto the surface of multi-modal registration (MSMAll) [22]. Surface registration based on multi-modal data was applied to task-fMRI images and fed into the statistical analysis.

#### 2.6.3. Statistical Analysis for Task fMRI

Statistical analysis of task-fMRI data was conducted using a three-level approach involving single-run level, individual-level, and group-level analyses. The individual-level analysis was an aggregation of the single-run level analysis, and both of these were performed using the FMRI Expert Analysis Tool (FEAT) [26,27,28] in the HCP pipeline [21]. The group-level analysis was performed using the FSL Permutation Analysis of Linear Models (PALM, version alpha135) [29].

In the single-run analysis, the preprocessed fMRI time series at each vertex in the task-fMRI data were fitted to a general linear model. Regressors that modeled blood oxygen level-dependent (BOLD) signals in response to the task events of Stroop and non-Stroop trials were included in the model. Surface spatial maps (such as one value per vertex in the brain) of the contrast of parameter estimates (COPEs) were computed, corresponding to the average activation during Stroop and non-Stroop trials. In the individual subject-level analysis, the average activation for session 1 from the first and second runs, session 2 from the third and fourth runs, and activation of the interaction between chocolate and time were computed based on the predefined contrast (Table 2). A group-level analysis was conducted on the COPEs obtained from the individual-level analysis with parceled surface images using PALM (10,000 permutations) [29]. The statistical threshold was set at −logP < 1.38 (corresponding to corrected *p* < 0.05).

### 2.7. Behavioral and Physiological Assessments

#### 2.7.1. Data Acquisition

##### Cognitive Task during fMRI

During the task-fMRI scan, participants performed the traffic light test [16,30], which is a modified version of the Stoop task comprising Stroop [31] and selective attention tasks. The task presentation comprised a traffic light, color name letters (blue or red in Japanese) superimposed on blue or red traffic lights, and traffic signs for pedestrians (right or left) and turns (right or left) above and below the traffic lights. Each trial was defined as a Stroop trial with a mismatch between the color name letter and background traffic light color, whereas a non-Stroop trial was defined as a match between them. The Stroop and non-Stroop trials were pseudo-randomly presented with a jittered 2–6 s inter-trial interval determined by a genetic algorithm [32]. The stimulus presentation was controlled by presentation software (Neurobehavioral Systems Inc., Berkley, CA, USA); the participants had to press the right or left button with their right index and middle fingers when a letter indicating blue or red in Japanese was presented, respectively, regardless of traffic signs for walkers and turns (BOLDscreen 32, Cambridge Research Systems Ltd., Rochester, UK).

##### Autonomic Nervous System Function

To assess the autonomic nervous system function, the Vital Monitor 302 system (VM302, Fatigue Science Institute, Osaka, Japan) was used to simultaneously measure the electrocardiogram and photoplethysmogram for 3 min while the participants sat quietly with their eyes closed. The recorded data were analyzed using the MemCalc software (GMS, Tokyo, Japan). A frequency analysis of R-R interval variation from electrocardiogram and a-a interval variation as the second derivative from accelerated plethysmography was performed using the maximum entropy method, which estimates the power spectral density from short time-series data and is suitable for studying changes in heart rate variability under different conditions for short periods [33,34]. The power spectrum resolution was 600 Hz. In the frequency analysis, power within the frequency range of 0.04–0.15 Hz and 0.15–0.4 Hz were calculated as low-frequency (LF) and high-frequency (HF) powers, respectively. The HF is mediated by the vagus nerve [35,36,37], whereas the LF is mediated by various sympathetic and vagal mechanisms [34,38]. Some review articles [39,40,41] mentioned that the LF reflects sympathetic nerve activity and used as a marker of sympathetic nerve activity. To stabilize the measurement, a 1 min pretest was performed before the 3 min main test. The main test was administered twice: before food intake and after the MRI scans (Figure 1).

##### Subjective Ratings

Subjective feelings were measured using a visual analog scale (VAS) for overall, mental, and physical fatigue, stress, boredom, sleepiness, motivation, relaxation, enjoyment, healing, concentration, and willingness before and after food intake (Figure 1). Participants were instructed to draw a line intersecting the 100 mm horizontal line at the location representing each of the feelings; the left and right ends of the VAS represented the lowest and highest values of each feeling, respectively.

### 2.8. Statistical Analysis

Cognitive function was analyzed based on the percentage of correct responses and average reaction times for both the whole trials and trial types. The autonomic nervous system function was analyzed for LF power, HF power, ratio of LF to HF (LF/HF), and the coefficient of component variance total power (ccvTP). Each subjective feeling was quantified by measuring the distance from the left end of the VAS to the position of the line drawn by the participants with an accuracy of 1 mm; the VAS scores ranged 0–100. Based on the predefined statistical analysis plan, values outside the mean ± 3 standard deviations range for mean reaction time and error rate in the cognitive tasks and LF/HF values in the autonomic nervous system function task were identified as error values and excluded from the statistical analysis. In this study, we focused on the contrast between sessions 1 and 2 for cognitive function and before and after chocolate consumption for autonomic nervous system function and subjective feelings. According to Rosnow and Rosenthal [42], in cases involving preplanned comparisons, it is possible to directly conduct multiple comparisons between specific groups without conducting an analysis of variance (ANOVA). Therefore, for statistical testing, we performed multiple comparison tests with Bonferroni correction for each chocolate condition. Paired *t*-tests were used for cognitive performance and autonomic nervous system function, and Wilcoxon signed-rank tests were used for subjective ratings on R software (version 4.2.2, R Foundation for Statistical Computing, Vienna, Austria).

## 3. Results

### 3.1. Participant Information

Statistical analysis for efficacy evaluation was performed using data from 26 participants who completed all tests and excluding those who met any of the exclusion criteria. The demographic data and statistical results are summarized in Table 3. The mean age and BMI were comparable between Groups A and B (age: t (24) = −0.54, *p* = 0.591; BMI: t (24) = −0.60, *p* = 0.551). The sex ratio of participants was also comparable between the groups (χ2 = 0.004, *p* = 0.951). Thus, no bias was observed in participant characteristics (age, BMI, or sex) between the groups with different orders of test food intake. The chocolate intake rate for each participant was 100% on all test days. No adverse events occurred during the study period.

### 3.2. Crossover Design Adequacy

To confirm the crossover design validity, we compared the mean reaction times and accuracy rates for all trial types in session 1 between the HC and LC intake groups. No significant difference was observed in mean reaction time (t (24) = −018, *p* = 0.86) and accuracy rate (t (24) = −0.89, *p* = 0.38), indicating that no biases were observed between groups.

### 3.3. Task-Related Brain Activities

**Figure 2:**
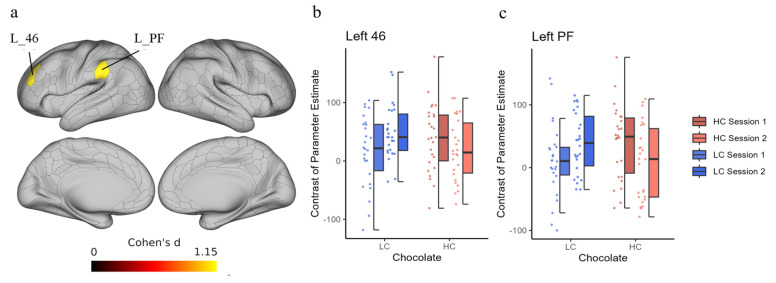
Interaction effect of time and chocolate in the Stroop trial. (**a**) These regions are depicted by predefined contrast by [LC_session 2–LC_session 1]–[HC_session 2–HC_session 1] in the Stroop trial. The two panels on the right show the contrast of parameter estimates (COPEs) in each condition at the left 46 (L_46) ROI (**b**) and left PF (L_PF) ROI (**c**) using dot plots and box charts. Labels of anatomical regions are according to the atlas provided in a previous study [43]. The color of the data points corresponds to the same condition as the color of the box plot. HC, high concentration; LC, low concentration; ROI, region of interest.

To identify differences in task-related brain activity after chocolate ingestion between the HC and LC intake groups in sessions 1 and 2, the preplanned contrast-based interaction effects were tested as follows: [LC_session 2–LC_session 1]–[HC_session 2–HC_session 1] for Stroop or non-Stroop trials. We found a significant interaction effect in the left 46 (dorsolateral prefrontal cortex; [DLPFC]) and left PF (supramarginal cortex including the posterior parietal cortex [PPC]) for the Stroop trials (Figure 2a). However, no significant interaction was observed in any brain region in the non-Stroop trials. Figure 2b,c show the COPEs for the Stroop trials in each condition. Delta COPE values (session 2−session 1) showed that, on average, the activity in session 2 was lower than that in session 1 after HC chocolate consumption, whereas the opposite pattern was observed after LC chocolate consumption (Table 4). In addition, higher delta values were observed in the LC condition than in the HC condition in the L_46 region in 22 out of 26 cases and in the L_PF region in 16 out of 26 cases.

### 3.4. Cognitive Function

**Figure 3:**
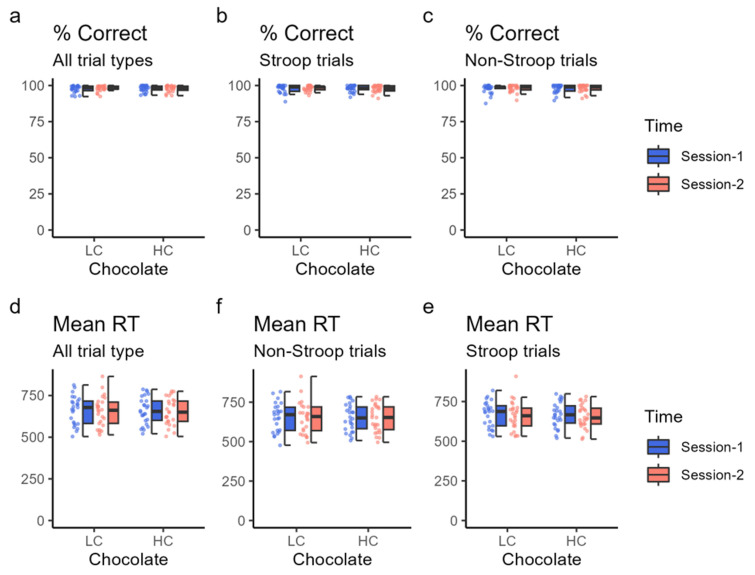
Cognitive task performance. Cognitive task performance was assessed based on the percentage of correct responses (% correct) and mean reaction time (RT). The top three panels show the percentage of correct response (%) in the “traffic light test” for all trials (**a**), Stroop trials (**b**), and non-Stroop trials (**c**) using dot plots and box charts. The bottom three panels show the average reaction time (RT) (in ms) in the “traffic light test” for all trials (**d**), Stroop trials (**e**), and non-Stroop trials (**f**). The color of the data points corresponds to the same condition as the color of the box plot. HC: high cacao concentration chocolate; LC: low cacao concentration chocolate.

Cognitive performance during task fMRI was assessed based on the mean reaction time and percentage of correct responses for whole, Stroop, and non-Stroop trials. The mean reaction times for session 2 were slightly shorter than those for session 1 in all, Stroop, and non-Stroop trials for both HC and LC but were not significant (*p* > 0.1 for all) (Table 5 and Figure 3). Furthermore, the percentage of correct responses was higher in session 1 than in session 2 for the Stroop trial when the HC chocolate was ingested; under all other conditions, the percentage of correct responses was higher in session 2. However, none of these differences were significant (*p* > 0.3 for all) (Table 5).

### 3.5. Autonomic Nervous System Function

**Figure 4:**
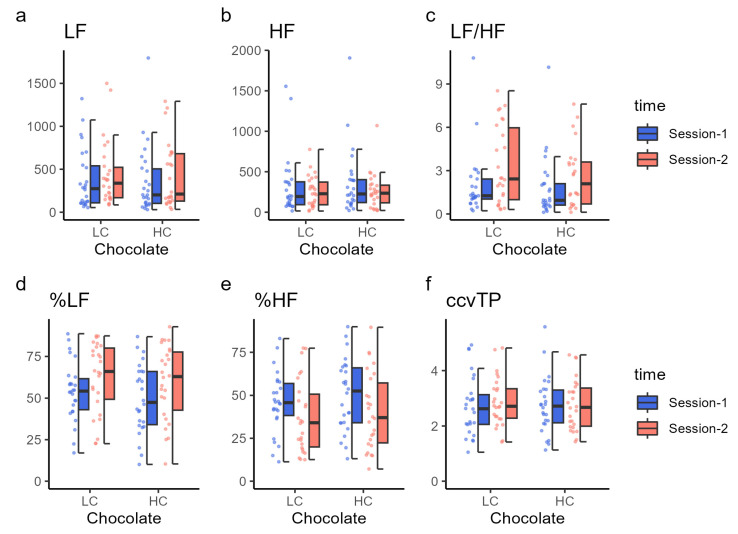
Autonomic nervous system function results. Each panel shows the (**a**) low-frequency power (LF), (**b**) high-frequency power (HF), (**c**) LF/HF ratio, (**d**) percent LF, (**e**) percent HF, and (**f**) ccvTP using dot plots and box charts. The color of the data points corresponds to the same condition as the color of the box plot. HC, high cacao concentration chocolate; LC, low cacao concentration chocolate.

Autonomic nervous system function was assessed based on LF power, HF power, their ratio (LF/HF), and the proportions of LF and HF (percent LF and HF) (Figure 4 and Table 6). LF, HF, total power (TP), and ccvTP did not significantly differ between sessions 1 and 2 when either HC or LC chocolate was ingested (Ps > 0.04, statistical threshold was set at 0.025, corresponding to 0.05 with Bonferroni correction). The LF/HF ratio increased significantly in session 2 compared with in session 1 for both HC (t (23) = 3.52, *p* = 0.002) and LC (t (24) = 2.45, *p* = 0.022) chocolate ingestion. Similarly, the percentages of LF and HF significantly changed after both HC (t (25) = 3.31, *p* = 0.003) and LC (t (25) = 2.65, *p* = 0.014) chocolate ingestion. The percentage of LF was higher in session 2 than in session 1, and the percentage of HF was lower in session 2 than in session 1, indicating that the balance between LF and HF power was dominated by LF in session 2.

### 3.6. Subjective Feelings

**Figure 5:**
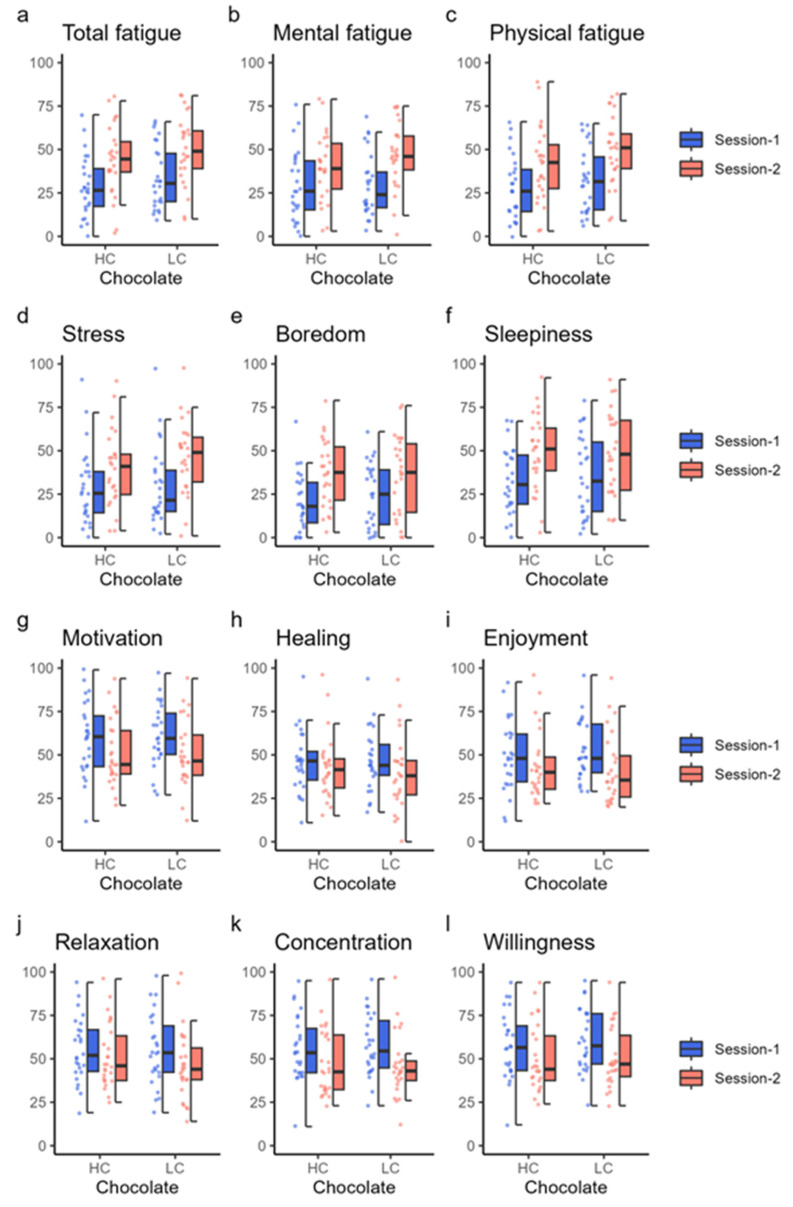
Subjective feeling results. Each panel shows the value of subjective feelings measured using a visual analog scale. The top six panels show the values of total fatigue (**a**), mental fatigue (**b**), physical fatigue (**c**), stress (**d**), boredom (**e**), and sleepiness (**f**) using dot and box charts. The bottom six panels show the values for motivation (**g**), healing (**h**), enjoyment (**i**), relaxation (**j**), concentration (**k**), and willingness (**l**). The color of the data points corresponds to the same condition as the color of the box plot. HC, high cacao concentration chocolate; LC, low cacao concentration chocolate.

Subjective feelings scored using the VAS are shown in Figure 5 and Table 7. Total fatigue, mental fatigue, physical fatigue, stress, boredom, and sleepiness significantly increased in session 2 compared with session 1 after both HC and LC chocolate ingestion; in contrast, motivation, enjoyment, concentration, and willingness were significantly decreased in session 2 compared with session 1 (Table 7). Healing and relaxation also decreased significantly in session 2 compared with session 1 when LC chocolate was ingested (V = 35.0, *p* = 0.003 for healing; V = 75.0, *p* = 0.011 for relaxation), but not significantly when HC chocolate was ingested.

## 4. Discussion

In this fMRI study, we observed that a single intake of HC chocolate enhanced the efficient use of cognitive resources to reduce the cost of brain activity during continuous and effortful tasks. When comparing brain activities during two consecutive cognitive task sessions with a short break in between, significant differences were observed between the HC and LC chocolate intake conditions in the differences in activation between session 1 and session 2 in the left BA46 (DLPFFC) and left BA40 (PF) ROIs. This indicates that brain activity in session 2 when HC chocolate was ingested was lower than that in session 2 when LC chocolate was ingested, although there were no significant changes in cognitive task performance.

Several recent studies have reported that cognitive efficiency can be increased by reducing the consumption of cognitive resources. For example, professional soccer players who play physical sports are reported to efficiently control their foot movements by significantly reducing the consumption of cognitive resources in the motor cortex of the brain [44]. Another study reported decreased brain activity in the DLPFC during attentional tasks after cognitive training [45]. Additionally, many previous fMRI studies have shown activation with less BOLD effects in quite discrete and task-dependent brain regions after acquiring skills in instrument [46] and computer game performance [47]. Thus, long-term training and upskilling result in energy-saving effects on neural activities. In this context, the novel finding of our study was that the cognitive resource-saving effect of HC chocolate intake was observed only 50–65 min after consumption.

In this study and a recent behavioral study [16], we used a traffic light task that requires high cognitive effort by combining a Stroop task [31] and a selective attention task. Stroop trials typically require attention to less salient stimulus attributes while ignoring more salient or automatically processed stimulus attributes. According to a meta-analysis by Cieslik et al. [48], the Stroop task primarily involves brain regions within the frontoparietal executive control network. Another study suggested that cognitive conflict due to color/word mismatch in Stroop trials activates the conflict monitoring in the anterior cingulate gyrus, which in turn activates executive control of the DLPFC to resolve the conflict. DLPFC activation may lead to increased attention in subsequent trials, resulting in better performance [49]. Moreover, the traffic light task requires additional executive control because selective attention is needed to suppress attention to interfering stimuli that are irrelevant to the task. When multiple tasks are simultaneously performed, each task’s performance decreases compared with when a single assignment is performed. This decrease may be caused by interference from other tasks that are simultaneously performed (dual-task interference effect), which reduces the cognitive resources directed toward the performance of the target task (task performance effect). Mizuno et al. [50] found that dual tasks increase parieto-frontal associations compared with single tasks, suggesting their association with demands for cognitive resources. Additionally, several studies have demonstrated a link between cognitive load/demand and brain activity, even in non-dual tasks, indicating that increased cognitive load in working memory tasks decreases performance but increases activity in brain regions associated with working memory [51]. Furthermore, cortical responses indicate that the same brain regions “work harder” rather than an increased network of brain regions [52]. Due to these specific features of the cognitive task, rather than a heavier load, we observed a difference in behavioral and fMRI data between HC and LC chocolate intake.

In this study that focused on task-related performance, LC chocolate intake decreased the overall correct response rates and correct response rates in Stroop trials in session 2 compared with session 1; however, HC chocolate intake did not have such a significant decrease in session 2, suggesting that HC chocolate consumption helps maintain performance and concentration for continuous and effortful cognitive tasks. The difference between the results from the behavioral and fMRI studies, especially for the decline of correct response rates in the second half of the LC chocolate intake group, may be explained by the different postures [53] during the studies: the sitting and supine positions in the behavioral and fMRI studies, respectively, and head restraint [54] in the fMRI study. Both factors are known to change regional cerebral blood flow and therefore alter task-related activation in the brain. Nevertheless, both results strengthened the effects of HC chocolate intake on brain function efficiency related to dual-cognitive tasks.

From a mechanistic point of view, we contemplated the reasons for HC chocolate mainly exhibiting such beneficial effects on neuronal activity- and energy consumption-saving and whether their actions derived from the effect on regional cerebral blood flow and synaptic plasticity, or a change in the efficiency of mitochondrial electron transport. Regarding the activation of brain regions, Magnore et al. [55] reviewed the activation of brain regions relevant to chocolate from various perspectives. Chocolate consumption stimulates various brain areas, especially chemosensory areas, including the insula, prefrontal region, and caudomedial and caudolateral orbitofrontal cortices [56]. fMRI studies have reported that the insular, human gustatory, and orbitofrontal cortices are central regions associated with taste [57,58]. Similarly, chocolate color modulates brain activity with a significant reduction in theta wave activity. This implies reduced attention and greater distraction [59]. Additionally, the sight of chocolate generated greater activation in chocolate cravers than in non-cravers in the medial orbitofrontal cortex and ventral striatum [60]. However, none of this evidence explains our results for the HC chocolate effect in the regional cognitive task-related brain activity-saving.

This study had several limitations. We emphasized the intake of high amounts of cacao polyphenols in chocolate formulations and designed a comparative study based on the dosage. In a systematic review, the effective dose of cacao polyphenols for a variety of health promotions was reported with cacao flavanol contents at approximately 500–900 mg/day [18]. However, the Japanese Ministry of Health, Labor, and Welfare recommends <200 kcal for snacks per day, and we could not recommend that consumers take more than 25 g of chocolate daily as a snack. Consequently, we did not design such a high dose of cacao flavanols in this study (approximately 160 mg/25 g of chocolate measured by our study group). Moreover, we could not use white chocolate, which does not contain cacao polyphenols, for the control condition because this would have been a cause of concern in terms of blinding, and the taste and components of white chocolate could have affected the evaluation, especially since it is difficult to reproduce the unique taste (bitterness and astringency) of dark chocolate with white chocolate. Thus, while it could have been possible to visually control the appearance of the chocolate, the taste would still have significantly compromised blinding. Additionally, milk-derived ingredients of white chocolate, such as milk protein, are known to affect cognitive function and mood states [61,62]. Another limitation is that the contributions of other HC chocolate ingredients such as caffeine and theobromine remain unclear in isolation from those of higher cacao polyphenols levels. Nonetheless, this study provides further evidence for the benefits of practical amounts of HC chocolate for brain activity, at least when compared to those of ordinary chocolate, as a daily supplement or snack. In the near future, more robust effects may be demonstrated when an increased intake of flavanols or other effective pure polyphenol(s) is tested.

## 5. Conclusions

Dark chocolate has been known to enhance brain activity related to cognitive performance, and the results of this fMRI study with adults in the late young and early middle-age groups suggested that a single intake of chocolate containing high concentrations of cacao polyphenols may contribute to increased brain functional efficiency during continuous and effortful tasks. Future studies investigating the mechanisms of the activity-saving and performance-maintaining effects of high-cacao polyphenols within a short time frame are required.

## Figures and Tables

**Table 1:** Test food ingredients.

	HC Chocolate	LC Chocolate
Color/Shape	Brown/Plate	
Amount (per test; g)	25.0 ± 1.0	
Energy (kcal)	131.9	138.5
Fat-free cacao content (g)	8.7	2.9
Total polyphenol (mg) *^1^	635.0	211.7
Epicatechin (mg) *^2^	30.4	10.5
Theobromine (mg) *^2^	200.0	66.7
Caffeine (mg) *^2^	20.0	6.7

*^1^ Folin–Ciocalteu method [19] using (−)-epicatechin (SIGMA Chemical Co., St. Louis, MO, USA) as a standard; *^2^ high performance liquid chromatography (HPLC) [20].

**Table 2:** Predefined contrast.

	HC Chocolate					LC Chocolate			
	Run-1	Run-2	Run-3	Run-4	Run-1		Run-2	Run-3	Run-4
Average of session 1 (HC)	1	1	0	0	0		0	0	0
Average of session 2 (HC)	0	0	1	1	0		0	0	0
Average of session 1 (LC)	0	0	0	0	1		1	0	0
Average of session 2 (LC)	0	0	0	0	0		0	1	1
Interaction	−1	−1	1	1	1		1	−1	−1
(chocolate × time)									

Predefined contrast for task functional magnetic resonance imaging analysis. HC, high concentration; LC, low concentration.

**Table 3:** Participant information.

	Group A	Group B	*p* Value
Number of participants (females/males)	14 (8/6)	12 (7/5)	0.951 ^a^
Mean age (±SD, years)	40.1 ± 4.5	41.2 ± 5.8	0.600 ^b^
BMI (±SD, kg/m^2^)	23.2 ± 3.1	24.0 ± 3.8	0.558 ^b^

^a^ *p*-values were obtained using the chi-square test. ^b^ *p*-values were obtained using the two-sample *t*-test. BMI, body mass index; SD, standard deviation.

**Table 4:** Descriptive statistics for contrast of parameter estimates.

	HC Chocolate			LC Chocolate		
	Session 1	Session 2	Delta	Session 1	Session 2	Delta
Left_46	40.3 ± 57.5	19.5 ± 52.7	−20.9 ± 50.6	17.9 ± 60.0	48.5 ± 45.7	30.5 ± 61.9
Left_PF	41.6 ± 58.2	8.2 ± 60.0	−33.4 ± 55.6	10.6 ± 57.8	38.5 ± 38.5	27.9 ± 64.9

Each value indicates average ± SD. Delta was calculated as the session 2 value−session 1 value. HC, high concentration; LC, low concentration; SD, standard deviation.

**Table 5:** Statistics for the cognitive function test.

	HC Chocolate							LC Chocolate						
	Average ± SD		Statistics: Session 2−Session 1					Average ± SD		Statistics: Session 2−Session 1				
	Session 1	Session 2	t	df	*p*	CI (Low)	CI (High)	Session 1	Session 2	t	df	*p*	CI (Low)	CI (High)
All trial types (Stroop and non-Stroop trials)														
Mean RT	659.5 ± 80.6	653.4 ± 80.0	−0.83	25	0.42	−21.18	0.05	662.2 ± 85.3	653.6 ± 89.8	−1.21	24	0.24	−23.29	6.04
% correct	97.9 ± 2.1	97.8 ± 2.1	0.08	25	0.89	−0.71	0.63	97.7 ± 2.3	98.0 ± 1.9	0.92	24	0.36	−0.38	0.99
Stroop trials														
Mean RT	666.7 ± 78.0	657.6 ± 73.7	−1.37	25	0.18	−22.65	4.59	670.4 ± 80.7	660.2 ± 90.4	−1.33	24	0.20	−26.10	5.62
% correct	97.8 ± 2.2	97.7 ± 2.3	−0.28	25	0.78	−1.01	0.77	97.8 ± 2.7	98.0 ± 1.9	0.38	24	0.71	−0.93	1.35
Non-Stroop trials														
Mean RT	652.2 ± 84.9	649.2 ± 87.4	−0.36	25	0.73	−20.70	14.61	653.9 ± 91.5	650.6 ± 100.3	−0.37	24	0.71	−21.53	15.04
% correct	97.9 ± 2.8	98.0 ± 2.6	0.11	25	0.91	−0.87	0.97	97.5 ± 3	98.0 ± 2.4	0.99	24	0.33	−0.46	1.31

Statistical threshold was set at *p* = 0.025, corresponding to *p* = 0.05 with Bonferroni correction. CI, confidence interval; df, degrees of freedom; RT, reaction time; SD, standard deviation.

**Table 6:** Statistics for autonomic nervous system function.

	HC Chocolate							LC Chocolate						
	Average ± SD		Statistics: Session 2−Session 1					Average ± SD		Statistics: Session 2−Session 1				
	Session 1	Session 2	t	df	*p*	CI (Low)	CI (High)	Session 1	Session 2	t	df	*p*	CI (Low)	CI (High)
LF	360.2 ± 391.8	428.3 ± 378.5	0.83	25	0.416	−101.56	237.89	375.3 ± 350.0	437.8 ± 377.8	0.95	25	0.354	−73.68	198.60
HF	362.4 ± 402.0	260.6 ± 218.1	−2.16	25	0.041	−199.1	−4.57	327.2 ± 375.7	254.3 ± 190.4	−1.34	25	0.193	−185.28	39.37
TP	722.6 ± 675.1	688.9 ± 523.1	−0.32	25	0.752	−251.55	184.17	702.5 ± 651.9	692.0 ± 481.4	−0.11	25	0.913	−205.32	184.35
LF/HF	1.8 ± 2.1	2.6 ± 2.2	3.52	23	0.002	0.51	1.98	2 ± 2.2	3.4 ± 2.7	2.45	24	0.022	0.20	2.35
ccvTP	2.7 ± 1.0	2.8 ± 0.9	0.22	25	0.828	−0.34	0.43	2.7 ± 1.1	2.9 ± 0.9	0.82	25	0.421	−0.21	0.49
% LF	49.7 ± 20.7	59.4 ± 22.3	−3.31	25	0.003	−15.81	−3.67	54.2 ± 17.7	62.4 ± 21.0	−2.65	25	0.014	−14.63	−1.83
% HF	50.3 ± 20.7	40.6 ± 22.3	3.31	25	0.003	3.67	15.81	45.8 ± 17.7	37.6 ± 21.0	2.65	25	0.014	1.83	14.63

Paired *t*-tests results. The statistical threshold was set at *p* = 0.025, corresponding to *p* = 0.05 with Bonferroni correction. Bold type indicates significance. CI, confidence interval of 95%; ccvTP, coefficient of component variance total power; df, degrees of freedom; LF, low frequency; HF, high frequency; SD, standard deviation; TP, total power.

**Table 7:** Statistics for subjective feelings.

	HC Chocolate						LC Chocolate					
	Average ± SD		Statistics: Session 2−Session 1				Average ± SD		Statistics: Session 2−Session 1			
	Session 1	Session 2	V	*p*	CI (Low)	CI (High)	Session 1	Session 2	V	*p*	CI (Low)	CI (High)
Total fatigue	28.6 ± 17.2	43.9 ± 20.2	320.5	<10^−3^	8.5	19.0	33.8 ± 18.0	49.8 ± 19.9	312.0	<10^−3^	9.0	0.0
Mental fatigue	29.2 ± 20.1	40.2 ± 19.4	286.5	<10^−3^	5.0	16.0	29.6 ± 18.1	46.4 ± 18.4	326.5	<10^−3^	8.0	24.0
Physical fatigue	28.1 ± 18.3	41.3 ± 21.7	317.0	<10^−3^	7.5	17.0	32.6 ± 18.6	49.1 ± 20.0	296.5	<10^−3^	9.5	23.5
Stress	29.2 ± 21.5	39.8 ± 21.8	260.0	0.002	4.5	16.0	29.6 ± 22.1	46.3 ± 21.0	277.0	<10^−3^	10.0	26.0
Boredom	20.4 ± 16.5	37.0 ± 19.3	296.0	<10^−4^	9.5	23.5	24.7 ± 18.3	36.0 ± 22.5	226.5	0.007	2.5	21.5
Sleepiness	32.8 ± 18.7	48.8 ± 21.7	320.5	<10^−3^	6.0	23.5	35.5 ± 23.3	48.6 ± 24.1	260.0	0.009	4.5	22.0
Motivation	59.4 ± 20.9	51.3 ± 19.0	49.0	0.004	−13.5	−3.5	61.0 ± 17.9	51.3 ± 19.3	41.5	0.001	−14.0	−5.0
Healing	46.2 ± 17.3	43 ± 18.3	86.5	0.693	−8.5	0.5	46.7 ± 17.7	39.3 ± 21.2	35.0	0.003	−13.5	−3.0
Enjoyment	49.4 ± 20.2	43.8 ± 19.5	76.0	0.020	−11.0	−1.0	52.1 ± 17.0	41.7 ± 19.9	52.0	0.003	−17.0	−3.5
Relaxation	55.2 ± 18.2	51.5 ± 18.5	86.5	0.127	−9.0	1.0	54.9 ± 20.4	46.6 ± 20.6	75.0	0.011	−15.5	−2.0
Concentration	55.6 ± 18.8	47.6 ± 19.0	62.0	0.004	−12.0	−3.5	57.8 ± 17.7	46.1 ± 17.8	49.5	0.001	−18.0	−4.5
Willingness	57.4 ± 19.3	50.4 ± 19.3	67.5	0.011	−10.5	−1.5	60.3 ± 18.1	50.7 ± 17.2	45.0	0.003	−17.0	−4.5

Wilcoxon signed-rank test results. The statistical threshold was set at *p* = 0.025, corresponding to *p* = 0.05 with Bonferroni correction. Bold type indicates statistical significance. CI, confidence interval of 95%; SD, standard deviation.

## Data Availability

The study data are available upon reasonable request from the corresponding authors.

## Supplementary Materials

The following supporting information can be downloaded from https://www.mdpi.com/article/10.3390/nu16010041/s1: Figure S1: Participant flowchart; Table S1: List of the inclusion and exclusion criteria.
