# Supplementary material for: Cacao Polyphenol-Rich Dark Chocolate Intake Contributes to Efficient Brain Activity during Cognitive Tasks: A Randomized, Single-Blinded, Crossover, and Dose-Comparison fMRI Study
Source: Nutrients. 2023 Dec 21;16(1):41. doi: 10.3390/nu16010041 (PMC10780455; doi:10.3390/nu16010041)
Supplement: Supplementary file 1 [file nutrients-16-00041-s001.zip › Cacao_fMRI_Nutrients_Supplementary tables_PF.pdf]

Supplementary Tables

Table S1. List of the inclusion and the exclusion criteria

**Inclusion criteria**	
1	Individuals who were fully explained the study, understood the study purpose and content, and had given their informed consent in writing of their own free will.
2	Healthy men and women aged of 30–49 years at the time consent was obtained.
**Exclusion criteria**	
1	Persons who consumed pharmaceuticals, quasi-drugs, or dietary supplements at least 5 days per week during the month prior to the SCR or planned to do so during the study period
2	Persons who have or had serious diseases such as cranial nerve disease, liver disease, kidney disease, cardiac disease, circulatory disease, malignant tumor
3	Persons who have had seizures (loss of consciousness, coma, convulsions due to neurological diseases
4	Persons with food allergies
5	Persons who could not consume bitter chocolate (dark chocolate)*
6	Persons who consumed excessive alcohol (average of 60 g/day or more)
7	Habitual smokers
8	Persons who worked day/night shifts, and those who worked night shifts during the period of the study
9	Persons who had extremely poor performance on the cognitive function test during the SCR.
10	Persons who could not complete the autonomic function test during the SCR.
11	Left-handed persons
12	Persons judged to be inappropriate as participants by the Principal Investigator or a Research Assigning Physician.

* Bitter (dark) chocolate was defined as chocolate with 40%–60% cocoa liquor without milk (dairy), chocolate with low sugar and dairy content and high bitterness, or low sugar chocolate with 70%–90% cocoa content. SCR, screening test.
