# Supplementary figures and images for: Cacao Polyphenol-Rich Dark Chocolate Intake Contributes to Efficient Brain Activity during Cognitive Tasks: A Randomized, Single-Blinded, Crossover, and Dose-Comparison fMRI Study
Source: Nutrients. 2023 Dec 21;16(1):41. doi: 10.3390/nu16010041 (PMC10780455; doi:10.3390/nu16010041)

Supplementary figures

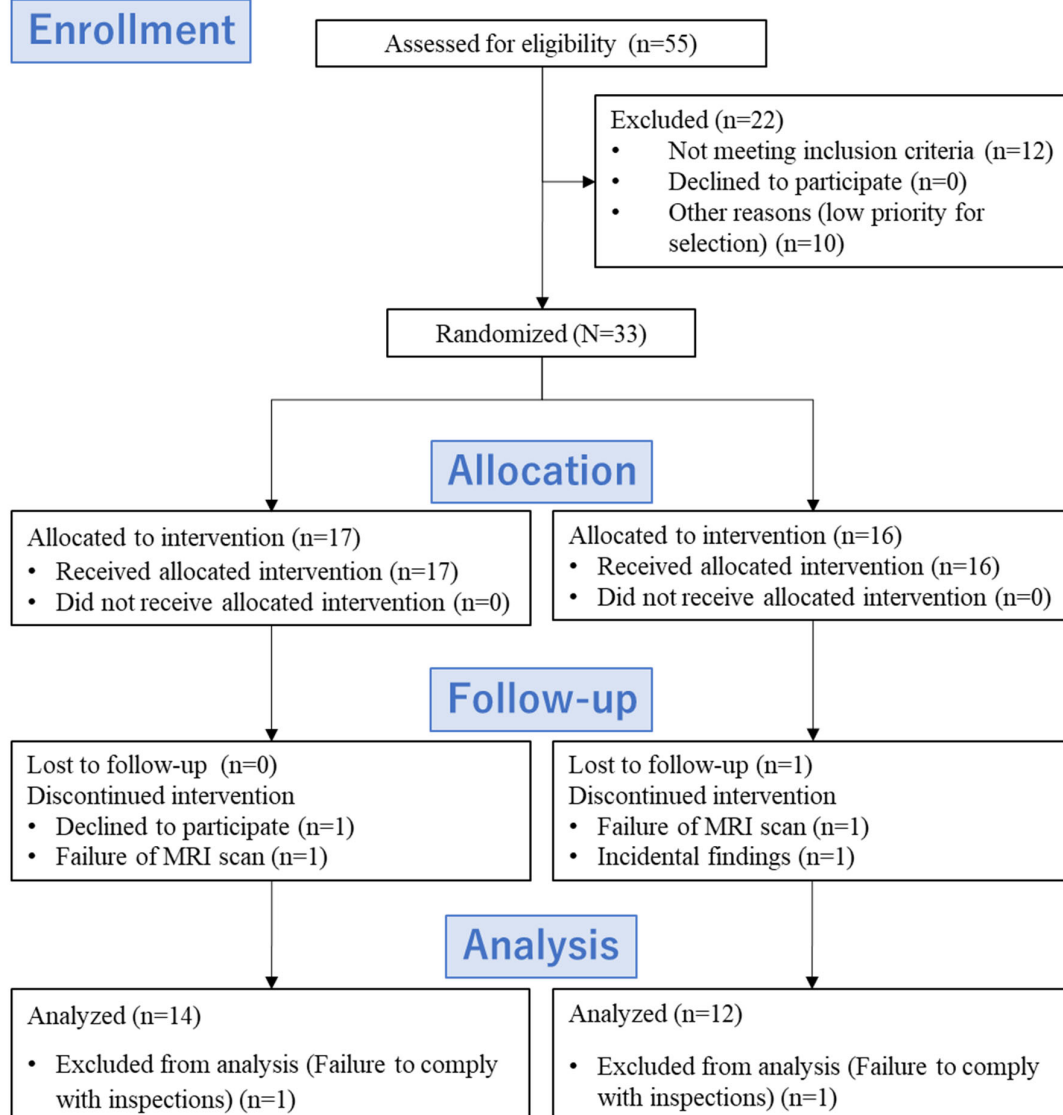

**Figure S1.** Participants' flowchart. MRI, magnetic resonance imaging.

Supplement: Supplementary file 1 [file nutrients-16-00041-s001.zip › Cacao_fMRI_Nutrients_Supplementary figures_PF.pdf]
